# Valence of emotions and moral decision-making: increased pleasantness to pleasant images and decreased unpleasantness to unpleasant images are associated with utilitarian choices in healthy adults

**DOI:** 10.3389/fnhum.2013.00626

**Published:** 2013-09-26

**Authors:** Martina Carmona-Perera, Celia Martí-García, Miguel Pérez-García, Antonio Verdejo-García

**Affiliations:** ^1^Department of Personality, Assessment and Psychological Treatment, University of GranadaGranada, Spain; ^2^School of Health Sciences, University of GranadaGranada, Spain; ^3^Centro de Investigación Mente, Cerebro y Comportamiento, University of GranadaGranada, Spain; ^4^Centro de Investigación Biomédica en Red de Salud Mental, University of GranadaGranada, Spain; ^5^Institute of Neuroscience Federico Olóriz, University of GranadaArmilla, Spain; ^6^Red de Trastornos Adictivos, Instituto Carlos III, University of GranadaSpain; ^7^School of Psychology and Psychiatry, Monash UniversityMelbourne, VIC, Australia

**Keywords:** moral-decision making, utilitarian choices, moral emotions, valence, arousal

## Abstract

Moral decision-making is a key asset for humans’ integration in social contexts, and the way we decide about moral issues seems to be strongly influenced by emotions. For example, individuals with deficits in emotional processing tend to deliver more utilitarian choices (accepting an emotionally aversive action in favor of communitarian well-being). However, little is known about the association between emotional experience and moral-related patterns of choice. We investigated whether subjective reactivity to emotional stimuli, in terms of valence, arousal, and dominance, is associated with moral decision-making in 95 healthy adults. They answered to a set of moral and non-moral dilemmas and assessed emotional experience in valence, arousal and dominance dimensions in response to neutral, pleasant, unpleasant non-moral, and unpleasant moral pictures. Results showed significant correlations between less unpleasantness to negative stimuli, more pleasantness to positive stimuli and higher proportion of utilitarian choices. We also found a positive association between higher arousal ratings to negative moral laden pictures and more utilitarian choices. Low dominance was associated with greater perceived difficulty over moral judgment. These behavioral results are in fitting with the proposed role of emotional experience in moral choice.

## INTRODUCTION

Moral decision-making is an essential asset for humans’ integration in social contexts. Emotional processes contribute to moral judgment by assigning affective value to the moral decision-making scenarios, thus guiding the distinction between acceptable and inacceptable behaviors (Haidt, 2001). The presentation of hypothetical scenarios involving moral violations typically generate subjective unpleasantness and increased arousal, which are thought to guide subsequent moral appraisals and decisions (Moll et al., 2002a; Ostrosky-Solís et al., 2003; Vélez-García et al., 2003; Harenski and Hamann, 2006). Moreover, the presentation of different types of moral stimuli, including moral-laden pictures (Moll et al., 2002a; Harenski and Hamann, 2006), moral statements (Moll et al., 2002b) or moral dilemmas (Greene et al., 2001, 2004; Heekeren et al., 2003, 2005; Blair, 2007) evoke significant changes in brain networks specialized in emotional processing, such as the ventromedial prefrontal cortex. Conversely, individuals with ventromedial prefrontal dysfunction (by virtue of psychopathology or brain lesions) and emotion processing deficits are typically more prone to endorse utilitarian choices, which maximize the aggregate welfare at the expense of the emotional implications of harming an innocent person (Greene et al., 2001; Koenigs et al., 2007; Carmona-Perera et al., 2012; Young et al., 2012).

According to the dual process theory (Greene, 2007; Greene et al., 2008) utilitarian choices are associated with higher order cognitive control, as illustrated by the impact of cognitive biasing factors on this type of judgments, including reasoning styles (Amit and Greene, 2012), cognitive load (Greene et al., 2008; Moore et al., 2008), priming reflection (Paxton et al., 2011), or attentional bias (Van Dillen et al., 2012). By contrast, deontological choices are preferentially supported by aversive emotional processing (Greene et al., 2001, 2004; Koenigs et al., 2007; Moretto et al., 2010; Carmona-Perera et al., 2013a,b). Recent studies have demonstrated that transient manipulation of specific emotions can bias moral decision-making toward utilitarian or deontological choices in response to moral dilemmas. Specifically, several studies have demonstrated that the induction of positively valenced emotions (e.g., happiness, humorous) favors the tendency to endorse utilitarian choices, whereas the induction of negatively valenced emotions (e.g, sadness, disgust) favors the tendency to endorse deontological choices (Wheatley and Haidt, 2005; Valdesolo and DeSteno, 2006; Schnall et al., 2008; Pastötter et al., 2012; Van Dillen et al., 2012). Complementarily, several studies have shown that the motivational tendency primed by the specific emotion induced is significantly associated with utilitarian vs. deontological choices in moral dilemmas. Specifically, the induction of approach-related emotions (e.g., anger) fosters the tendency to endorse utilitarian choices, whereas the induction of avoidance-related emotions (e.g., disgust) fosters the tendency to endorse deontological choices (Harlé and Sanfey, 2010; Ugazio et al., 2012). Although these studies elegantly show how transient manipulations of particular emotions can bias moral decision-making in different directions, considerably less is known about how more stable individual differences in emotional experience (in response to a range of emotionally competent stimuli) are associated with decision-making patterns in moral vs. non-moral scenarios. The Lang bio-informational model of emotion assumes that individual differences in emotional experience can be reliably and efficiently tracked using the subjective responses to emotional stimuli on three relevant aspects of emotion: valence (pleasantness/unpleasantness of the experience), arousal (activation generated by the experience), and dominance (degree of control that one is able to exert over the emotional experience induced; Greenwald et al., 1989; Lang et al., 1993). In this dimensional system, categorical emotions are quantitatively represented; for example, anger would be linked to high unpleasantness, high arousal and high dominance, whereas fear would be linked to high unpleasantness, high arousal but low dominance.

In this study we aimed to investigate whether individual differences in emotional experience, based on the Lang model, are associated with individual differences in moral decision-making patterns, as measured by a battery of moral (and non-moral) dilemmas (Greene et al., 2001). Specifically, we examined whether individual differences in subjective reactivity to affective stimuli is specifically associated with moral (vs. non-moral) decision-making in healthy adults, and whether individual variations in the valence, arousal and dominance subjective emotional ratings are associated with specific utilitarian vs. deontological choice patterns. Based on the previous literature, we hypothesize that (1) individual differences in emotional experience will be specifically correlated with decision-making in moral but not in non-moral scenarios; (2) subjective ratings indexing greater unpleasantness, high arousal and low dominance emotional experience will be associated with predominantly deontological choice patterns, whereas subjective ratings indexing lower experience of unpleasantness, low arousal and high dominance will be associated with predominantly utilitarian choice patterns.

## MATERIALS AND METHODS

### PARTICIPANTS

The sample consisted of 95 healthy adults (49 males and 46 females). All participants were of European-Caucasian origin and were recruited from local community and recreational centers during the first semester of 2011 through flyers-based advertisement and word-of-mouth communication. Eligibility criteria were defined as follows: (i) to be literate enough to ensure reading comprehension, and correctly complete the tests; (ii) not having lifetime use of illegal drugs in more than five occasions or current or past diagnoses of substance dependence (with the exception of nicotine); (iii) not having history of head injury or neurological disorders; (iv) and not having clinically significant psychiatric symptoms. The Interview for Research on Addictive Behaviour (IRAB; Verdejo-García et al., 2005) was used to assess compliance with the absence of drug use/dependence criterion, and the Symptom Checklist-90-Revised (SCL-90-R; Derogatis, 1977) was used to assess compliance with the absence of significant psychiatric symptoms criterion. The sample had a mean (standard deviation) of 49 years-old (10.67) and 18 years of education (2.38). Socioeconomic status was assessed through occupation prestige and mean family income (through self-reports). We classified the participants into three socioeconomic categories: low level (17.9%), average level (63.2%) and high level (17.9%). None of these demographic variables affected moral decision-making (all *p* > 0.05).

### INSTRUMENTS

#### Emotional experience task

We used a set of 40 picture stimuli extracted from the International Affective Picture System (IAPS; Lang et al., 1988) and other sources such as the internet. Based on the IAPS norms (Lang et al., 1988), we defined four picture categories or conditions of interest: (i) neutral (10 pictures displaying landscapes, household objects), (ii) pleasant (10 pictures displaying sexual and radical sports scenes), (iii) unpleasant non-moral laden (10 pictures displaying accident-related casualties or mutilations), and (iv) unpleasant moral laden (10 pictures displaying poverty or one to one violence scenes). Since moral content is not addressed in the IAPS norms, we conducted a pilot study (*n* = 83 undergraduate students) to evaluate “perceived moral content” in an initial pool of 22 images with suitable contents for the unpleasant moral laden category. The 10 images with higher “perceived moral content” ratings (>7.5 in a 1–10 range) were finally included in this (iv) category. As a further check the 40 images included in the emotional experience task were also evaluated for “perceived moral content” by the study sample (*n* = 95), and we confirmed that the 10 images included in this category significantly differed from those included in the other categories on “perceived moral content” [*F*(3, 282) 721.20, *p* < 0.001]. The main dependent measure for each of the picture categories were the subjective ratings of valence (from 1 –unpleasant– to 9 –pleasant–), arousal (from 1 –relaxed– to 9 –aroused–), and dominance (from 1 –dominant– to 9 –dominated–). The responses were recorded using the Self-Assessment Manikin (SAM; Lang, 1980). As dependent variables we used the mean of valence, arousal and dominance scores for each of the four categories of pictures.

#### Moral decision-making task

We used a subset of 32 hypothetical dilemmas extracted from Greene battery (Greene et al., 2001). The original Greene battery was adapted to Spanish language through a back-translation process. The ensuing items were evaluated using Rasch analysis to obtain a briefer construct-valid measure of moral decision-making (Carmona-Perera et al., under review). We used the calibration and item fit tests to remove redundant and low quality items impacted by commonly confounding variables outside moral decision-making (e.g., socio-demographic factors). We also excluded those moral dilemmas that fell at the tails (>95%) of the deontological or utilitarian response distributions, since they are less likely to constitute an actual decision dilemma. The final 32-item Spanish version has demonstrated adequate psychometric properties (Cronbach’s alpha = 0.78, Spearman Brown coefficient = 0.76; Carmona-Perera et al., under review). This task is composed by eight non-moral dilemmas involving a rational decision without moral content (e.g., to travel by train or bus given certain time constraints, or to buy a new camera or to have your old camera repaired for the same price), and 24 moral dilemmas which concern the appropriateness of moral violations for a higher benefit (e.g., smothering a baby to save a group of people, or throwing a dying person into the sea to keep a lifeboat of survivors afloat). These moral dilemmas involve different degrees of emotional salience based on the extent of personal involvement and the ensuing severity of harm (Greene et al., 2001, 2004). Therefore, the task included both Personal dilemmas (16 items) which involve higher emotional salience and Impersonal dilemmas (8 items) which involve lower emotional salience. Participants were asked to provide “choice” (affirmative vs. negative) and “perceived difficulty” responses (from 1 –low– to 10 –high–). For moral dilemmas affirmative answers were considered “utilitarian” (e.g., to kill someone to save a group of people), and negative answers “deontological” (e.g., to refuse the harmful action regardless the aggregate well-being). For non-moral dilemmas affirmative answers were considered “efficient” (e.g., to travel by the fastest transport to arrive on time), and negative answers “non-efficient” (e.g., to travel by the preferred transport despite off to arrive late). The proportion of affirmative choices and the mean of perceived level of difficulty for moral and non-moral scenarios were computed as main dependent variables.

### PROCEDURE

This study was approved by the Ethics Committee for Human Research of the University of Granada. Before testing, all participants were informed about the study protocols and they signed a written informed consent to certificate their voluntary collaboration. The information sheet included the following information: “We are interested in exploring how you make decisions in relation to a set of moral and non-moral hypothetical scenarios, and how you experience emotions in relation to a set of affective pictures. We will ask you to decide whether you would accept or refuse to take a proposed action concerning moral and non-moral scenarios. In a separate task, we will ask you to report your subjective emotional experience in response to both pleasant and unpleasant stimuli.” To describe each SAM scale we used the standardized guidelines of Lang et al. (2001). Participants were assessed individually in a single session that lasted approximately 90 min. The emotional experience and the moral decision-making tasks were administered in computerized format using two different orders, such that half of the sample performed first the dilemmas and then the pictures and half of the sample did it in the reverse sequence. In the emotional experience task categories were presented in a counter-balanced order across participants. In all cases, each picture was presented during 6 s, followed by a 2 s black screen with a fixation cross. Participants were instructed to stare at the picture and rate their emotional experience using the SAM scales of valence, arousal and dominance, with no time limits established. In the moral dilemmas task the different subsets of dilemmas (moral personal, moral impersonal, non-moral) were also presented in a counter-balanced order. Each was presented through three subsequent computer screens. The first screen described the dilemma (presentation); the second screen presented the response options and requested the choice (decision-making), and the third screen presented the difficulty scale and requested the perceived difficulty rating. Each screen continued with no time limit as the participants read and responded to the dilemmas.

### STATISTICAL ANALYSIS

We used repeated-measures ANOVAs to test the main effects of picture categories on subjective valence, arousal and dominance ratings in the emotional experience task, and of type of dilemma on the affirmative choices and perceived difficulty in the moral decision-making task. Pairwise Bonferrroni *post-hoc* tests were used to examine specific effects driven by the different picture categories and types of dilemmas. To test our main assumptions, we conducted Pearson product-moment correlation analyses between the valence, arousal, and dominance ratings to the images included in the different picture categories, and the choices and difficulty ratings to the moral and non-moral dilemmas. The Bonferroni correction was used to adjust the significance levels of correlation coefficients for multiple comparisons (Curtin and Schulz, 1998). Results are presented reporting the corrected *p* values.

## RESULTS

### SUBJECTIVE REACTIVITY TO EMOTIONAL STIMULI

Results showed the expected significant differences between the valence, arousal and dominance ratings evoked by the images grouped in the different picture categories (see **Table 1**). Pairwise comparisons showed significant effects in all contrasts, with the exception of the contrast between unpleasant non-moral laden and unpleasant moral laden categories, which had similar valence, arousal and dominance ratings. Moreover, the unpleasant pictures (non-moral and moral) yielded higher unpleasantness and arousing ratings, and lower dominance ratings, than all other conditions.

**Table 1:** Descriptive scores, ANOVA and *post-hoc* comparisons for emotional valence, arousal, and dominance.

Subjective reactivity	Picture ctegories, Mean (SD)				*F*	*Post-hoc *Non-significant (*p*)
	Neutral (1)	Pleasant (2)	Unpleasant non-moral laden (3)	Unpleasant moral laden (4)
Valence	5.65 (0.86)	7.47 (0.90)	1.94 (0.72)	1.96 (0.73)	143.97*	3 = 4 (*p* = 1.000)
Arousal	3.97 (1.22)	5.82 (1.60)	7.46 (0.86)	7.60 (0.83)	213.18*	3 = 4 (*p* = 0.511)
Dominance	8.42 (0.90)	7.49 (1.43)	5.26 (2.27)	5.07 (2.35)	148.67*	3 = 4 (*p *=1.000)

*F *value associated to the tests of within-subjects effects (Huynh-Feldt correction); *p value <0.01

### DECISION-MAKING AND DIFFICULTY RATINGS TO MORAL AND NON-MORAL DILEMMAS

ANOVA analyses showed significant differences between moral and non-moral dilemmas in terms of affirmative choices [*F*(1, 94) = 593.82, *p* < 0.001], and difficulty [*F*(1, 94) 346.34, *p* < 0.001]. Moral dilemmas yielded less affirmative choices (*M* = 58.72, SD = 13.78) and higher perceived difficulty (*M* = 4.08, SD = 1.38) than non-moral dilemmas (*M* = 97.24, SD = 5.82, and *M* = 1.58, SD = 0.64, respectively). We also found significant differences between personal and impersonal moral dilemmas on utilitarian choices [*F*(1, 94) 765.56, *p* < 0.001] and difficulty ratings [*F*(1, 94) 51.81, *p* < 0.001]. Personal dilemmas yielded less utilitarian choices (personal: *M *= 28.62, SD = 20.01; impersonal: *M* = 88.82, SD = 14.29) and higher perceived difficulty (personal: *M* = 4.69, SD = 1.70; impersonal: *M* = 3.48, SD = 1.50).

### ASSOCIATION BETWEEN SUBJECTIVE REACTIVITY TO EMOTIONAL STIMULI AND UTILITARIAN CHOICES AND DIFFICULTY RATINGS TO DILEMMAS

**FIGURE 1:**
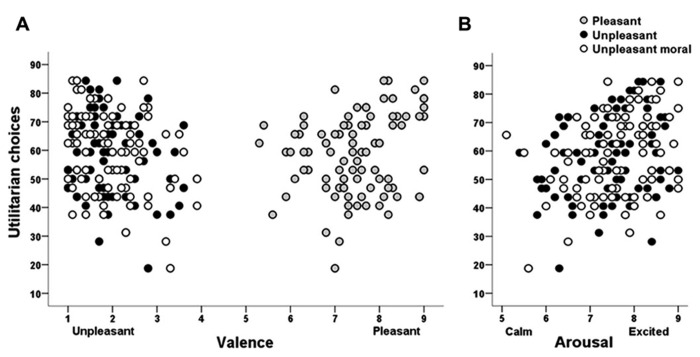
**Dispersion graph of correlation between proportion of utilitarian choices in moral dilemmas and subjective reactivity of valence (A)** and arousal **(B)** to affective stimuli.

Results showed that the subjective ratings evoked by the emotional stimuli were specifically associated with decision-making in moral, but not in non-moral, scenarios. The proportion of affirmative (utilitarian) choices in moral dilemmas (merging both personal and impersonal dilemmas) correlated with both valence and arousal ratings (see **Figure 1**). However, we found non-significant correlations between the proportion of affirmative (efficient) choices in non-moral dilemmas and the subjective ratings of valence, arousal or dominance (all *p* ≥ 0.170). Separate correlations between personal or impersonal moral dilemmas and the emotional experience task failed to show significant effects in all picture categories for valence (*p* ≥ 0.181), arousal (*p* ≥ 0.096) and dominance ratings (*p* ≥ 0.235).

Significant correlations between moral (personal and impersonal) decisions and emotional experience indicated that moral choices were associated with valence ratings to both unpleasant (moral: *r* = -0.29, *p* = 0.016; non-moral: *r* = -0.26, *p* = 0.043) and pleasant images (*r* = 0.26, *p* = 0.047); experiencing less unpleasantness in response to unpleasant images (both moral and non-moral), and more pleasantness in response to pleasant images were associated with more utilitarian choices (see **Figure 1A**). Moral choices were also associated with arousal ratings to unpleasant moral laden images (*r* = 0.34, *p* = 0.004); higher arousal responses correlated with more utilitarian choices (see **Figure 1B**). The perceived difficulty ratings to the moral dilemmas were negatively correlated with dominance ratings across the moral and non-moral negative picture categories (unpleasant non-moral, *r* = -0.26; *p* = 0.043; and unpleasant moral, *r* = -0.29;* p* = 0.016). Conversely, moral choices or difficulty ratings failed to correlate with the perceived moral content of the images.

## DISCUSSION

The main findings of this study are the following: (1) individual differences in self-reported emotional experience correlate with decision-making in moral scenarios, but not in non-moral scenarios; (2) lower experience of unpleasantness to both moral and non-moral unpleasant images and higher experience of pleasantness to pleasant images are associated with utilitarian choice patterns; (3) higher experience of arousal (specifically in response to moral laden images) are associated with more utilitarian choices; and (4) lower dominance over emotions is significantly associated with higher perceived difficulty to make decisions in moral scenarios. In agreement with our initial hypotheses, these findings support the specific association between emotional experience and moral decision-making, and support the notion that diminished experience of unpleasantness favors utilitarian choice patterns. The association between higher arousal to unpleasant moral laden pictures and utilitarian choices, and between low dominance and higher moral difficulty were not originally predicted and may warrant further research.

In agreement with previous findings, we showed that decision-making in healthy populations is sensitive to the impact of moral vs. non-moral content scenarios (Moll et al., 2001; Harenski and Hamann, 2006; Tassy et al., 2013; Van Bavel et al., 2013), and to the impact of personal vs. impersonal involvement within these moral scenarios (Greene et al., 2001, 2004; Moretto et al., 2010; Koenigs et al., 2007; Carmona-Perera et al., 2013a). Difficulty of judgment may also contribute to describe the emotional weight attached to these choices. For example to push a button to kill someone (low emotional salience) is considered easier than to push a person to the train tracks (high emotional salience). Therefore, participants demonstrated sensitivity to moral content, and to the degree of emotional salience associated with this content.

Correlation analyses showed that moral-related patterns of choice (including both personal and impersonal dilemmas) correlate with subjective emotional experience, at difference with non-moral related decisions. Because separate consideration of personal and impersonal dilemmas did not result in significant correlations with emotional experience, our results can only speak of the association between moral-related decisions and emotional experience. In this respect, our findings are in agreement with those of previous studies that have demonstrated associations between the processing of moral (vs. non-moral) contents and emotional reactivity (Moll et al., 2001; Harenski and Hamann, 2006; Tassy et al., 2013; Van Bavel et al., 2013). The direction of the significant correlations between higher subjective valence ratings and higher proportion of utilitarian choices in moral dilemmas are in agreement with the specific role of emotional processes in moral decision-making (Greene et al., 2001; Haidt, 2001). The dual process model of moral judgment posits that a decreased sensitivity to the negative emotional input attached to moral violations may foster utilitarian choice patterns (Greene, 2007; Greene et al., 2008). Therefore, it is expectable that those individuals with less ability to experience unpleasantness are more prone to endorse utilitarian choices. The findings can also be theoretically accounted by the “undoing hypothesis,” which proposes that positive moods can “undo” the cognitive and physiological effects of negative emotions, thus decreasing experience of unpleasantness and increasing utilitarian biases (Fredrickson et al., 2000; Fredrickson and Branigan, 2005). These findings are also in agreement with a plethora of previous evidence demonstrating that induction of positive emotions reliably bias moral decision-making toward utilitarian patterns (Pastötter et al., 2012; Valdesolo and DeSteno, 2006).

In partial disagreement with our initial hypothesis (lower arousal associated with utilitarian choices) we found a positive correlation between higher arousal ratings to unpleasant moral laden pictures and higher proportion of utilitarian choices. These findings can be accounted by the inverted U-shaped association between arousal and decision-making, whereby moderate levels of arousal are optimal to process the emotional input that is relevant for decision-making, but too much or too little arousal become disrupting (Miu et al., 2008). Specifically, it has been demonstrated that high levels of arousal are associated with reduced ability to detect the relevant aspects of emotional input in the context of emotion regulation for dilemmas-solving (Blair et al., 2012). Therefore, we tentatively suggest that higher arousal sensitivity may be associated with greater influence of the emotional information that is irrelevant to address the moral dilemmas. Alternatively, these findings could be interpreted in the context of attentional control models of emotions, which postulate an attentional interference effects due to a higher arousal levels (Schimmack and Derryberry, 2005; Jefferies et al., 2008). Decreased attentional control has been recently linked to utilitarian choices (Van Dillen et al., 2012). Therefore, attentional process may also account for these findings, playing a moderator role between emotional experience and utilitarian choices.

An additional interesting finding was the association between lower dominance over emotions and higher perceived difficulty to decide about the moral dilemmas. Previous cognitive neuroscience studies have associated individual differences in emotional regulation with moral decision-making, identifying that lower emotional control increase the difficulty to decide, since the individual is driven into a more exhaustive appraisal process (Harenski et al., 2009; Bartels and Rips, 2010; Koven, 2011). Lower dominance ratings are associated with lower emotional control in the perceived situation (Bradley and Lang, 1994), such that our results agree with the notion that lower emotional control associates with more complex (more highly difficult-perceived) appraisal processes.

In summary, we provide novel evidence about the association between subjective emotional experience and moral decision-making in a community sample. The strengths of the study include the use of a representative community sample from the healthy population, the use of well-validated quantitative measures of emotional experience and moral decision-making, and the potential relevance of our findings for clinical implications. Because we show that variations in emotional experience, but not in subjective perceptions of moral content, are associated with utilitarian biases, we reason that the interventions for individuals with moral judgment problems should focus on training and shaping emotional response, rather than working on the “rules” characterizing moral violations. These type of emotional interventions may be useful to restore social decision-making in patients with acquired brain injuries (Koenigs et al., 2007; Moretto et al., 2010), psychopathy (Blair, 2007; Young et al., 2012) or drug addictions (Carmona-Perera et al., 2012; Khemiri et al., 2012). Our results should be also interpreted in the context of its relevant limitations. First, personal and impersonal moral dilemmas (differing on emotional salience) did not differentially correlate with emotional experience. Hence, future studies are warranted to explore whether our findings can be replicated in more heterogeneous samples allowing further variance within personal and impersonal categories. Second, since emotional input impacts not only on moral choice but also on a range of other decision-making processes (Paulus and Yu, 2012) future studies are also warranted to determine whether reported associations apply only to utilitarian vs. deontological moral decision-making choices, or to a wider spectrum of decision-making scenarios. An additional limitation is the choice to base the emotional measurement only on subjective responses, disregarding complementary physiological or external behavioral indices (Lang et al., 1993) that should be included in future studies; and the non-measurement of some potential moderators of the link between emotion and moral decision-making – e.g., cognitive processes (Greene et al., 2008; Moore et al., 2008; Paxton et al., 2011; Amit and Greene, 2012; Van Dillen et al., 2012), personality traits (Bartels and Pizarro, 2011), or desirability to social and experimental demands (Lumer, 2010; Hess and Kotter-Grühn, 2011; Caravita et al., 2012).

## Conflict of Interest Statement

The authors declare that the research was conducted in the absence of any commercial or financial relationships that could be construed as a potential conflict of interest.
