# Erratum: Valence of emotions and moral decision-making: increased pleasantness to pleasant images and decreased unpleasantness to unpleasant images are associated with utilitarian choices in healthy adults

**DOI:** 10.3389/fnhum.2014.00050

**Published:** 2014-02-13

**Authors:** Martina Carmona-Perera, Celia Martí-García, Miguel Pérez-García, Antonio Verdejo-García

**Affiliations:** ^1^Department of Personality, Assessment and Psychological Treatment, University of GranadaGranada, Spain; ^2^School of Health Sciences, University of GranadaGranada, Spain; ^3^Centro de Investigación Mente, Cerebro y Comportamiento, University of GranadaGranada, Spain; ^4^Centro de Investigación Biomédica en Red de Salud Mental, University of GranadaGranada, Spain; ^5^Institute of Neuroscience F. Oloriz, University of GranadaArmilla, Spain; ^6^Red de Trastornos Adictivos, Instituto Carlos IIIUniversity of Granada, Spain; ^7^School of Psychology and Psychiatry, Monash UniversityVictoria, Australia

**Keywords:** arousal, moral emotions, moral-decision making, utilitarian choices, valence

We have noticed that we made an incorrect interpretation of the correlation between valence ratings to unpleasant pictures and proportion of utilitarian choices (Figure 1, Panel A, left side). The Figure is correct but our interpretation was wrong. We originally interpreted that decreased unpleasantness was associated with higher utilitarism, but the data points to the opposite direction: increased unpleasantness is associated with higher utilitarism. This interpretation error impacts the third line of the published title (“*decreased* unpleasantness to unpleasant images” should be “*increased* unpleasantness to unpleasant images”), line 11 of the abstract (“*less* unpleasantness to negative stimuli” should be “*more* unpleasantness to negative stimuli”), and one line in the Results subsection “ASSOCIATION BETWEEN SUBJECTIVE REACTIVITY TO EMOTIONAL STIMULI AND UTILITARIAN CHOICES AND DIFFICULTY RATINGS TO DILEMMAS”: the sentence “experiencing *less* unpleasantness in response to unpleasant images (both moral and non-moral), and more pleasantness in response to pleasant images were associated with more utilitarian choices (see Figure 1A)” should be “experiencing *more* unpleasantness in response to unpleasant images (both moral and non-moral), and more pleasantness in response to pleasant images were associated with more utilitarian choices (see Figure 1A).” Moreover, in the first paragraph of the Discussion, finding (2) “*lower* experience of unpleasantness” should be “*higher* experience of unpleasantness.” Therefore, while we originally argued that findings “support the notion that *diminished* experience of unpleasantness favors utilitarian choice patterns,” the correct interpretation is that “*increased* experience of unpleasantness favors utilitarian choice patterns.”

During replication of the whole set of statistical analyses in SPSS v. 20, we have noticed additional errors in the Results section. The *F*-value of the within-measures comparison of valence ratings should be 1143.97 instead of 143.97. Several *r*-values from correlation analyses were incorrect and the Bonferroni correction to *p*-values was incorrectly applied. Therefore, here, we present the correct *r-values* and exact *p*-values of these analyses, and we clarify the Bonferroni-corrected alpha level to interpret results. The exact *p*-value of the correlation between moral choices and valence ratings to unpleasant moral images should be 0.004 instead of the published value (0.016). The exact *p*-value of the correlation between moral choices and valence ratings to unpleasant non-moral images should be 0.011 instead of the published value (0.043). The *r* and exact *p*-values of the correlation between moral choices and valence ratings to pleasant images should be *r* = 0.21 and *p* = 0.040 instead of the published values (*r* = 0.26, *p* = 0.047). The *r* and exact *p*-values of the correlation between moral choices and arousal ratings to unpleasant moral images should be *r* = 0.31 and *p* = 0.002 instead of the published values (*r* = 0.34, *p* = 0.004). The *r* and exact *p*-values of the correlation between difficulty ratings and dominance ratings to unpleasant non-moral images should be *r* = −0.24 and *p* = 0.020 instead of the published values (*r* = −0.26, *p* = 0.043). The *r* and exact *p*-values of the correlation between difficulty ratings and dominance ratings to unpleasant moral images should be *r* = −0.31 and *p* = 0.002 instead of the published values (*r* = −0.29, *p* = 0.016). When applying a Bonferroni correction taking into account 2 types of dilemmas and 4 image conditions, the alpha value for all the correlations reported must be established at 0.006. Three previously unreported correlations concerning impersonal moral dilemmas were detected utilizing these parameters: a negative correlation between unpleasantness ratings to unpleasant pictures and utilitarian choices (*r* = −0.33, *p* = 0.001), a negative correlation between unpleasantness ratings to moral-laden pictures and utilitarian choices (*r* = −0.37, *p* < 0.001) and a positive correlation between arousal ratings to moral-laden images and utilitarian choices (*r* = 0.32, *p* = 0.002).

To recap, in the published article we reported four main findings, namely: (1) individual differences in self-reported emotional experience correlate with decision-making in moral scenarios, but not in non-moral scenarios; (2) *lower* experience of unpleasantness to both moral and non-moral unpleasant images and higher experience of pleasantness to pleasant images are associated with utilitarian choice patterns; (3) higher experience of arousal (specifically in response to moral laden images) are associated with more utilitarian choices, and (4) lower dominance over emotions is significantly associated with higher perceived difficulty to make decisions in moral scenarios. In this Erratum, we note that finding (2) should be reappraised as follows: *higher* experience of unpleasantness, *mainly to moral unpleasant images*, is associated with utilitarian choice patterns. This association was observed for the combined measure of utilitarian choices to moral dilemmas, and for utilitarian choices to impersonal dilemmas. Moreover, the correlation between pleasantness ratings and utilitarian choices would not survive a Bonferroni correction. Our revamped conclusion about the direction of correlations is that increased negative valence, increased arousal and lower dominance to emotional stimuli correlate with utilitarian choices. This interpretation cannot be easily framed in the dual-process theory (paragraph 3 of the Discussion) which has been useful to explain utilitarian choices in clinical populations, but actually fits better with the notion that certain types of negative emotions are linked to utilitarian choices in healthy populations.

